# Supplementary material for: Experimental study on chorus emission in an artificial magnetosphere
Source: Nat Commun. 2024 Feb 15;15:861. doi: 10.1038/s41467-024-44977-x (PMC10869741; doi:10.1038/s41467-024-44977-x)
Supplement: Supplementary file 2 — Description of Additional Supplementary Files [file 41467_2024_44977_MOESM2_ESM.pdf]

File name: Supplementary Movie 1

Description: Audio data of Whistler mode chorus emissions observed in the artificial magnetosphere RT-1. In the hot-electron high-β plasma (where β is the ratio of plasma pressure to magnetic pressure) generated in the dipole magnetic field of RT-1, chorus emissions are spontaneously excited. The fluctuations consist of intermittent electromagnetic waves around several tens of MHz with clear temporal frequency variations, and rather broad electrostatic fluctuations around 110 MHz, in proximity to the local electron cyclotron frequency. To make these audible, the audio file is played back at 1/10,000 of the original speed. This adjustment is necessary because the magnetic field strength of RT-1 is approximately ten thousand times stronger than that of the geospace, causing the observed Whistler wave frequencies to significantly surpass the human audible range. Whistler mode chorus emissions are ubiquitous phenomena that commonly occur in the geometry of a dipole magnetic field, despite notable differences in field strength and spatial scales between laboratory and space environments.
